# Design and acceptance assessment of a digital product passport for recycled and natural aggregate concrete elements

**DOI:** 10.1371/journal.pone.0347562

**Published:** 2026-04-20

**Authors:** Annkathrin Sinning, Sophie Würger, Wei Guo, Wan Li, Torben Miny, Tobias Kleinert, Martin Classen, Sabine J. Schlittmeier, Jan Bielak, Christian Böffel

**Affiliations:** 1 Institute of Structural Concrete, RWTH Aachen University, Aachen, Germany; 2 Work and Engineering Psychology, RWTH Aachen University, Aachen, Germany; 3 Cluster of Excellence CARE, TU Dresden and RWTH Aachen University, Aachen, Germany; 4 Chair of Information and Automation Systems for Process and Material Technology, RWTH Aachen University, Aachen, Germany; Xi#39;an University of Architecture and Technology, CHINA

## Abstract

The construction sector accounts for a substantial share of global greenhouse gas emissions, making effective strategies for the reuse and recycling of building materials indispensable. However, relevant information may be lost over the relatively long use phase of buildings. Digital Product Passports (DPPs) offer a standardized means of preserving and communicating product information across the life cycle and may therefore also be applied in the construction sector to facilitate reuse and recycling of building components, even after service lives exceeding 50 years. This paper presents the results of a first, interdisciplinary study that (i) develops a DPP for concrete elements using the Asset Administration Shell (AAS) and (ii) experimentally evaluates how DPP-presented information shapes consumer perceptions of recycled aggregate concrete (RAC) versus natural aggregate concrete (NAC) stair elements. In a scenario-based vignette experiment (*N* = 83), participants evaluated eight DPP mock-ups in which material (RAC vs. NAC), environmental impact (low vs. high), and structural performance (high vs. low) were systematically manipulated. Participants indicated their willingness to pay, perceived environmental value, perceived functional risk and product preference for each DPP. Repeated-measures ANOVAs showed robust main effects of material and environmental impact on perceived environmental value, and main effects of material and structural performance on perceived functional risk. Willingness to pay and product preference were higher for RAC than NAC, for low versus high environmental impact, and for high versus low structural performance. Overall, RAC was perceived as more environmentally valuable but also as riskier than NAC, even when objective environmental and structural indicators were held constant. The results indicate that DPP design should account for target-group-specific interpretation and potential biases in processing technical and sustainability information, to better support resource-efficient decision-making in the construction sector.

## Introduction

In 2023, 34% of the worldwide greenhouse gas emissions were attributed to the construction industry, making it the highest contributor [1]. A huge part of these emissions result from concrete still being the most widely used building material. Next to high CO_2_-emissions in cement production, natural resources such as gravel and sand are required, leading to depletion of natural resources. At the same time, the global annual amount of construction and demolition waste is estimated to two billion tons [1]. The recycling and reuse of mineral construction waste is indispensable for circular economy and in 2024, recycled materials accounted already for 18 percent of the materials used in construction in Europe [1]. This paper focuses on one specific key material in circular construction, recycled aggregate concrete (RAC). Furthermore, the concept of a Digital Product Passport (DPP) is introduced for building elements to facilitate reuse and recycling at the end of life of a building product. To overcome the present linear economy, the concept of circular economy aims to reuse structural or material components [2], e.g., as RAC. Due to the long service life of a building structure, required information for reuse or recycling at end of life (EOL) is often not preserved. The concept of a DPP is considered a potential solution in collecting and exchanging product-related data. A DPP contains comprehensive information on a product throughout its entire life cycle, including also information on potential EOL strategies [3]. Standardized DPPs for building materials or components, however, have not yet been established. In Germany, a first attempt for a standardized DPP for buildings is made by the German Sustainable Building Council (DGNB). The DGNB Building Resource Passport (BRP) [4] shall provide information on the material composition of a building with the respective information for establishing a circular demolition. The BRP considers the whole building, whereas templates for single building components are provided, but these are for informational use only and have not yet been integrated in the calculation of the life cycle analysis (LCA).

The successful implementation of sustainability measures such as the use of RAC largely depends on how these measures are subjectively perceived by consumers. The greatest barrier to the widespread purchase and use of sustainable alternatives is the lack of readily available information for consumers to base their decisions on [5–7]. As the DPP aims to provide comprehensive information about the entire life cycle, including sustainability aspects, it is essential for enhancing consumer acceptance and encouraging the adoption of sustainable building materials. Previous research shows how disclosing sustainability information is needed [8], and can improve the acceptance of a sustainable product, resulting in an increased purchase intention [7,9–11]. Therefore, it can be hypothesized that providing information on the environmental impact of sustainable building materials via a DPP should thus increase their acceptance.

However, despite the majority of sustainability literature assuming rational and linear consumer decision making [12], product perception is rarely objective. Instead, consumers and decision-makers are subject to typical constraints of human information processing. Decision-making can be influenced by so-called heuristic thinking [13], meaning the use of mental shortcuts or cognitive “rules of thumb”, which can lead to biased judgments [14]. These biases are present in laypeople and experts alike [15–17]. For example, the negative footprint illusion, where the addition of environmentally friendly items to a set of conventional items is perceived as reducing the set’s environmental impact despite actually increasing it, was found to be as prevalent among experts as among novices [15]. There is also evidence of a recycling bias within the general population, indicating an overestimation of the positive effects of recycling in the context of waste management [18]. Even though consumers generally report positive attitudes towards recycled materials, they commonly do not actually purchase them [19], as indicated by a reduced willingness to pay compared to the conventional product [10,20–22]. This is described as an attitude-behavior gap, where positive attitudes towards sustainable products do not correspond to sustainable behavior [23,24]. In one study [27], results revealed that, depending on the product category, sustainability could even be a liability. Correspondingly, some studies show how a performance risk is perceived for sustainable building materials [9,25], posing another barrier for their adoption. The increased risk perception for sustainable building materials should be reduced by additionally providing information about the product’s structural performance and safety through technologies such as DPP.

Despite previous studies on human perception of recycled products, there is a lack of understanding the human factor within the context of recycled materials in buildings. Considering the human factor at early development stages is integral for the adoption not only of sustainable building materials, but also for the implementation of innovative technologies such as a DPP. It is necessary to investigate how the presentation of objective data with a DPP is perceived by potential consumers to further guide the development of circularity within the building sector.

In a joint research project, the feasibility of a DPP for building components is investigated using prototypes of DPPs for concrete elements. We chose the relatively simple staircase element as a demonstrator for this study, since staircases can be considered a closed unit, which not only enables independent technical evaluation, but is also familiar to even laypersons. The aims of the paper are: 1. to discuss fundamental requirements of a DPP for concrete elements and 2. to explore the potential of such a DPP to promote sustainable decisions in the construction sector through comparison of natural aggregate concrete (NAC) and RAC elements. Therefore, in the first step, the input data for the prototype DPP is defined. As a standardized DPP for batteries already exists, this Battery Pass [26] is taken as an example and adapted for the use case of construction elements. The concept of the Asset Administration Shell (AAS) is used for modelling the DPP, since, thereby, interconnection and aggregation of DPPs becomes possible. This modelling approach is consistent with prior DPP research that operationalizes the AAS as the underlying information model to structure life-cycle data for interoperability and circular economy use cases, e.g., multi-stakeholder DPP concepts, circularity-oriented DPP data models, and implementations in secure data-sharing architectures and remanufacturing scenarios [27–32]. Finally, the perception of prototypical concrete elements presented with DPPs is investigated in an exemplary consumer group.

## State of the art

### Recycled aggregate concrete as a contribution to Circular Economy

**Fig 1:**
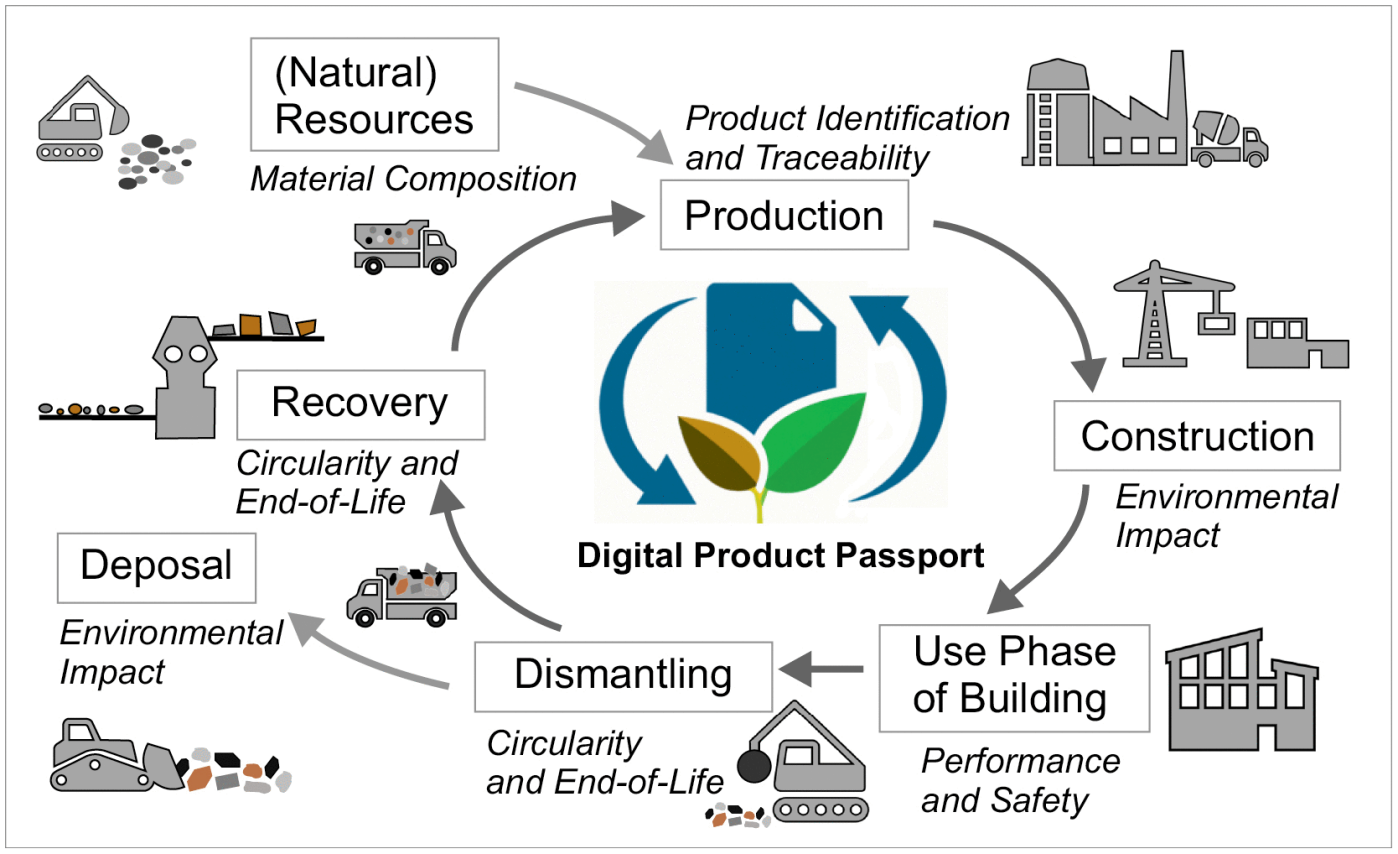
DPP Lifecycle. Life cycle of a building product and integration of main categories in DPP.

The main goal of a Circular Economy (CE), a concept that currently gains weight in research and practice [33], is to preserve natural resources, reduce waste and extend the life span of an item [34]. As a substitute for natural aggregates, construction and demolition waste (CDW) can be processed into smaller fractions and be used as recycled aggregates (RA). The quality of RA depends strongly on the quality of the CDW and potential sorting or separation steps before crushing [35]. If mixed CDW is delivered to the construction waste processor, pre-sorting is necessary, especially to remove hazardous materials that would hinder a later use as RA in structural concrete [35,36]. The more classification steps are introduced in the process, the more fractions suitable for concrete production can be obtained. On the other hand, more classification or sieving steps signify higher energy-consumption. In 2020, RA made up 13.2% of the demand for aggregates for concrete production in Germany [33], while more than half of the RA was used in road construction or other non-structural applications [33], which signifies a “down-cycling” [37]. With regard to CE and the future shortage of locally available natural resources, the reuse in building construction is direly needed. Fig 1 shows the life cycle of a building product and the potential of information integration in a DPP. A predatory competition between the use of RA in (new) building construction and established applications with a down-cycling should be avoided [38]. Instead, the total recycling rate of CDW needs to be enhanced [39] to ensure that the approximately 425 million tons generated in one year in the EU which are considered suitable for a processing to RA are reused in the sense of CE [37,40]. To facilitate the classification process, data from a DPP could be used to gain knowledge on the original composition of the demolished elements.

### Data availability and the potential of Digital Product Passports (DPPs)

In 2020, a Circular Economy Action Plan (CEAP) was adopted by the European Commission [41]. In this context, a DPP can enhance the traceability of a product and its components as well as its environmental impact (Fig 1). This also includes information on potential reuse of individual elements. It is assumed that environmental factors will play a crucial role in the near future and will therefore have a greater impact in the decision-making process. Direct comparability of the environmental impact including the life cycle analysis (LCA) of products throughout the complete life cycles and their recycling potential can be realized using the data stored in DPPs. Another challenge when comparing LCA is that often different datasets for the same materials are provided in databases such as ÖKOBAUDAT [42]. Therefore, the conclusions drawn may differ if different datasets are used, hence, potentially leading to misguided decisions [43]. In this context, a DPP seems to be an adequate tool to reduce inconsistencies in data and to minimize insecurities due to necessary assumptions and attribution of data from generalized databases. In this paper, a prototype of a DPP for reinforced concrete elements is presented considering the actual LCA data of the materials and including necessary information for an easier recyclability at EOL. The data stored in the DPP can also be used to determine an Urban Mining Index [2,44] that highlights the recyclability or reuse potential at EOL.

### Asset Administration Shell as an enabler for the DPP

The pragmatic shift towards CE emphasizes the need for enhanced transparency and traceability of products throughout their life cycle [45]. The DPP, emerging as a crucial instrument in this context, offers a comprehensive digital representation of products that facilitates data exchange and interoperability among stakeholders [41]. One of the first prototypical implementations of DPP is the Digital Product Passport for Industry 4.0 (DPP4.0), which is developed by the German Electro and Digital Industry Association (ZVEI) in collaboration with the Industrial Digital Twin Association (IDTA) and various stakeholders in the industrial sector [46]. The core components of DPP4.0 include the Identification Link defined by the IEC 61406 series (IEC 61406−1 [47] and IEC 61406−2 [48]), which specifies the relevant principles and restrictions for the product identifier, and the AAS outlined in the IEC 63278 standard [49], which structures product data in a modular format.

Beyond DPP4.0, multiple research contributions have already applied the AAS concept to DPP modelling in different contexts. Pourjafarian et al. propose a multi-stakeholder DPP architecture based on the AAS to support lifecycle-wide information sharing across stakeholders [32]. Gleich et al. demonstrate an AAS-based DPP implemented as a Gaia-X service, using AAS submodels to cover lifecycle information and enable standardized exchange via service interfaces [31]. Kühn et al. develop and validate an AAS-based DPP data model explicitly aimed at circular-economy requirements and cross-industry applicability [30]. Further, Abdel-Aty et al. evaluate DPP usage for remanufacturing in a case study using the AAS [29], and Engel et al. propose a minimal DPP data model aligned with regulatory compliance while ensuring interoperability through standardized AAS submodel templates [28]. Furthermore, the upcoming European standard EN 18216 identifies the AAS as a compatible protocol for the exchange of DPP data, further supporting the direct modelling of DPPs based on the AAS framework [27].

The AAS serves as a standardized digital interface for assets within the Industry 4.0 framework. It encapsulates relevant digital information associated with an asset, effectively functioning as its digital twin [50]. The core features of the AAS related to this work include [51]:

- Unique Identification: With global unique identifiers, each identifiable element is individually recognized within a digital environment.
- Interoperability: By utilizing elements like semantic ID and concept description, the AAS promotes interoperability among various systems and manufacturers
- Modular Structure: Submodels contain specific datasets related to different aspects of assets, like technical specifications, operational data, and life cycle information.

Based on a uniform and standardized metamodel, the AAS ensures consistency and interoperability of asset data across various systems and platforms, which is essential in modern industrial environments and business processes. Further, IDTA and related working groups specify application programming interfaces (API) and serializations for different data formats (e.g., JSON or XML) to enable manipulation, communication and exchange of AAS [52]. Utilizing such standardized framework and the supporting software tools, assets from different manufacturers can be easily integrated into digital ecosystems along entire value chains and value networks [53], enabling seamless communication and exchange of asset data. By aligning asset data with universal information models, AAS promotes efficiency and reduces the potential for data misinterpretation [54].

IDTA working groups, comprising experts from asset providers, users and regulators, further specify Submodel templates based on the AAS metamodel. The Submodel templates serve as standardized blueprints for representing aspect-specific information of an asset within the AAS environment, such as technical specifications, operational data, or life cycle information [55]. The Submodel templates specify generic attributes, which may directly apply to one or multiple domains.

In the concrete industry, the AAS can be utilized to create digital representations of concrete elements, considered as digital twins. They capture data for a concrete element in a comprehensive manner, especially the data required by the EU’s DPP regulations [56]. Furthermore, utilizing standardized Submodel templates within the DPP brings several advantages, including improved interoperability across platforms and organizations, reduced complexity through a structured development framework, enhanced compliance with industry standards and regulations, and scalability to incorporate future technological advancements. Recent developments emphasize the integration of AAS with data spaces [57], enabling scalable data sharing across companies and industries using the AAS as a common exchange format. Thus, implementing the DPP4.0 for concrete elements enables stakeholders to enhance quality control, optimize maintenance schedules, and improve sustainability assessments [53].

To ensure these benefits persist over the year lifespan of a building, the AAS framework inherently provides a strategy for long-term data viability. Its foundation as an international standard (IEC 63278) prevents technological obsolescence. Furthermore, the use of semantic IDs, as previously discussed, ensures that data remains not just syntactically readable but also semantically comprehensible for future systems. This technical robustness, combined with continuous governance by organizations like the IDTA, provides the stability required for the DPP to function throughout the asset’s entire life cycle.

## DPP4.0 for concrete elements

### Methodology for DPP implementation

IDTA recommended six Submodel templates to facilitate the creation of a DPP4.0 [58]. However, our analysis of the attributes defined in these Submodel templates reveals that they cannot be directly applied to concrete elements. This is due to the significant differences between the attributes embedded in these templates and those required for concrete elements, which are specified across multiple standards, such as “type of concrete” or “shear resistance” according to [59,60]. In the present research, we undertook a systematic approach to utilize the existing Submodel templates to meet the specific requirements of the concrete industry:

1. Analysis and Definition of Required Attributes: We began by collecting, analyzing and defining the attributes necessary for a DPP tailored to concrete elements. These attributes are further grouped into several categories and subcategories for overview and clarity. As a result, a data attribute list is generated at this step.
2. Comparison with existing IDTA Submodel Templates and ECLASS [61]: Next, we assessed the existing Submodel templates provided by the IDTA. This step involved a thorough comparison of the identified attributes with those represented in the standard Submodel templates, allowing us to pinpoint areas where the existing models aligned or diverged from our needs. The remaining attributes were matched with ECLASS (V14) [61] to identify a standardized property.
3. Development of DPP4.0 Prototype Template: We modeled the prototype using the grouped attributes resulting from Step 1. Each attribute category (see Fig 3) forms a distinct Submodel, which consists of attribute subcategories modelled as SubmodelElementCollections (SMCs). From the comparative analysis in the previous step, we identified the attributes that are semantically equal to those in IDTA and ECLASS. The resulting AAS serves as a DPP4.0 template for creating DPPs for concrete elements.
4. Creation of DPP4.0 Instances: For two example concrete elements (made from RAC and NAC), we collected data following the required attributes. A dedicated python module was developed to automatically transform the data from the data attribute list to corresponding DPP4.0 instances according to the DPP4.0 template. The resulting DPPs were made available in a dedicated repository. To link the DPPs with the physical concrete elements, QR codes compliant with the IEC 61406 standard were generated using the URLs of DPPs in the repository.
5. Generation of DPP Mock-Up: To enable the psychological study, a mock-up demonstrating the DPP data relevant for target user group was created.

This approach enabled us to benefit from established DPP4.0 frameworks while creating a prototype specifically designed to address the unique characteristics and requirements of concrete elements. As a passive, optical, and low-cost marker, a QR code is inherently more durable and less susceptible to technological obsolescence than dynamic data carriers like NFC tags over a building lifespan. The resulting structure maintains compatibility with broader DPP initiatives while providing the detailed documentation capabilities needed for the construction industry. In this project, an AAS template and two AAS prototypes were created. Details are elaborated in the following Section.

### Implementation and results

**Fig 2:**
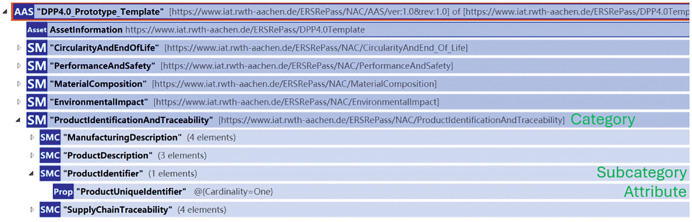
DPP4.0 Structure. Structure of the DPP4.0 prototype template (displayed with Eclipse AASX Package Explorer).

As specified in the recommendations for the development of DPPs, the EU Battery Passport [62] serves as a reference for the development of a DPP for concrete elements. In this study, 64 relevant attributes were identified and described in the data attribute list analogous to the Battery Passport Long Attribute List [63]. However, all of the attributes are only static results based on available information. The original raw data sources are not stored in the list. These attributes are hierarchically categorized. In total, five main categories are defined: (1) Product Identification and Traceability, (2) Material Composition, (3) Environmental Impact, (4) Performance and Safety, as well as (5) Circularity and End-of-Life. Each main category is further divided into multiple subcategories, with each attribute assigned to a specific subcategory. For instance, the attribute “Product Unique Identifier” is assigned to the subcategory “Product Identifier”, which belongs to the category “Product Identification and Traceability” (see Fig 2).

**Fig 3:**
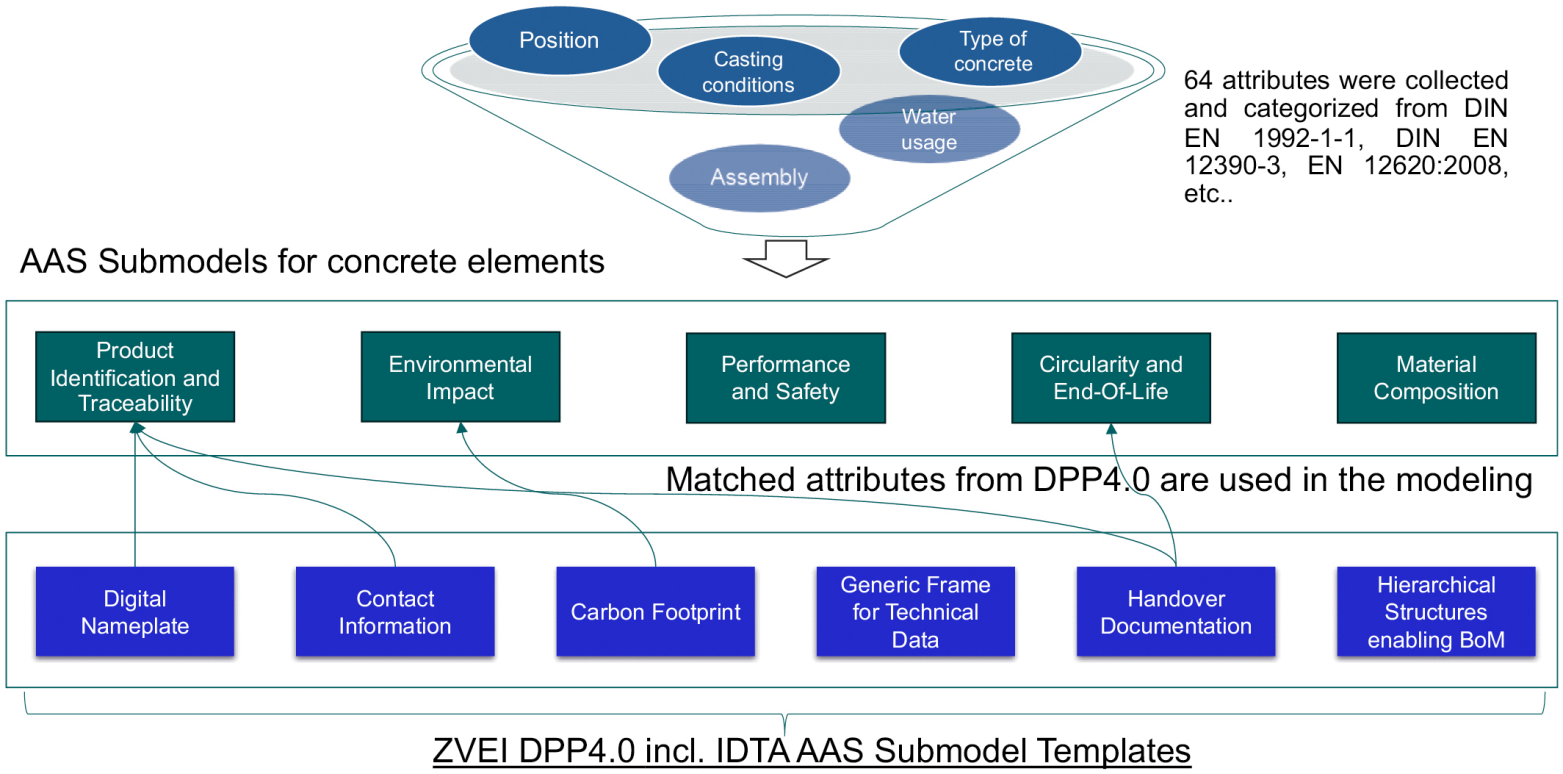
Integration of AAS and DPP4.0. Overview of the developed AAS for concrete elements and its relationship to the DPP4.0.

To ensure interoperability and alignment with industry standards, we conducted a thorough examination of the published Submodel templates provided by IDTA. The recommended Submodel templates for DPP4.0 include IDTA 02006-2-0 Digital Nameplate for Industrial Equipment [64], IDTA 02002-1-0 Submodel for Contact Information [65], IDTA 02023-0-9 Carbon Footprint [66], IDTA 02003-1-2 Generic Frame for Technical Data for Industrial Equipment in Manufacturing [67], IDTA 02011-1-0 Hierarchical Structures enabling Bills of Material [68], and IDTA 02004-1-2 Handover Documentation [69]. The referenced Submodel template and property were used in the AAS prototype for concrete elements (see Fig 3). For instance, we utilized the “PCFCO2eq” property from the Carbon Footprint Submodel template to represent our “CO2 emissions” attribute. For attributes that have no counterpart in existing Submodel templates, we created new ones according to idShort complaint format, such as “type of element” to “TypeOfElement”. For selecting the semanticId of the attribute, standard vocabularies (such as ECLASS [61] or CDD [70]) should be used if available. However, only attributes within Digital Nameplate, Carbon Footprint, and Contact Information submodels directly corresponded to concrete element properties in our work. The Handover Documentation submodel was specifically utilized to define and structure the documents to be preserved.

For all attributes, data types and cardinalities are specified based on their definitions and the required quantity. If the data type is of the file type, it is modeled using the Handover Documentation template. For attributes that need to express their content as a list, a Submodel Element Collection (SMC) is created to represent them as a group. For example, the “Assembly” is usually available in a pdf document (Data type ◊ File), while the “origin of materials” can be described by a list of material names with their origin.

**Fig 4:**
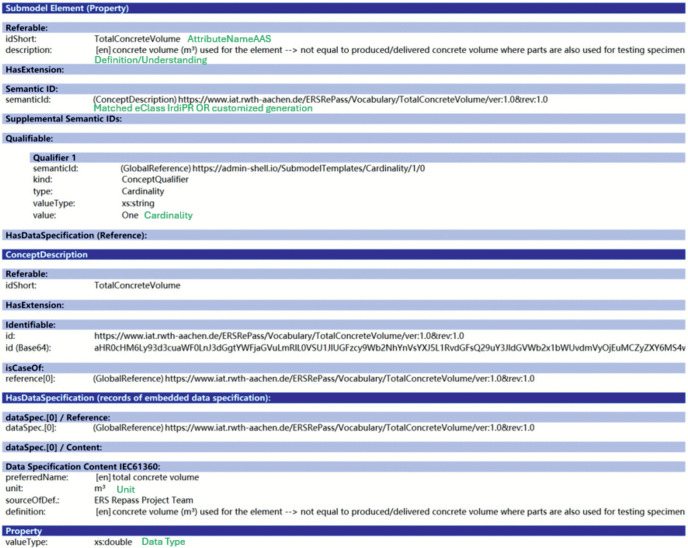
Example DPP attribute. Detailed presentation of an example attribute (displayed with Eclipse AASX Package Explorer).

To automatically generate standard-compliant AAS-based DPPs, we followed an established approach for generating AAS prototypes from scratch [71], which utilizes the Eclipse BaSyx Python SDK [72] and ECLASS [61]. First, the attributes are matched to the primary keys (IrdiPR) of the identified properties in ECLASS to ensure maximal semantic interoperability. Based on this, the attributes are structured in an intermediate Comma Separated Values (CSV) format, which is predefined by the toolkit, and filled with actual concrete elements data. The prepared dataset is then automatically transformed to DPPs of the exemplary concrete elements, according to the DPP4.0 template. SemanticIds are assigned to the SubmodelElements using either the matched IrdiPRs from ECLASS or the ones defined in the project. The internal content and structure of a SubmodelElement are illustrated using the example of *TotalConcreteVolume* in the DPP prototype as shown in Fig 4, with green text annotations indicating the specific attributes included from the data attribute list.

After creating the DPP template, two lists with attribute values (one for NAC and one for RAC) were used to generate the corresponding DPP instances.

The generated DPPs were shared among the project participants using a DPP repository implementing an HTTP Server [73] provided by the Eclipse BaSyx Python SDK. Thus, each DPP is assigned with a URL, which is used to create an IEC 61406-compliant QR code. With the generation of the QR code, the foundational elements of the DPP4.0 concept were successfully implemented. Furthermore, the DPPs can be accessed using the aas-python-http-client [74] for developers or viewed in a user-friendly interface for end-users through connecting the Eclipse Mnestix Browser [75] to the DPP repository. In addition, the DPPs can also be accessed through the QR code, which is attached to the corresponding concrete elements and their documentation. With such supporting tools, a digital ecosystem for creating, using and exchanging DPPs can be progressively achieved.

### Lifecycle data update during dismantling and recovery

Considering the long service life of building components and their integration into circular value chains, the AAS-based DPP is not conceived as a static dataset but as a continuously updatable digital twin. The AAS framework enables lifecycle-oriented data management through versioning mechanisms, unique asset identification, and standardized APIs defined by the IDTA [49,52]. During the use phase, operational and maintenance information can be appended via creation of dedicated Submodels for, e.g., Maintenance or Condition Monitoring. In the Dismantling phase, additional attributes such as dismantling date, residual structural performance, contamination status, and documentation of selective demolition can be added or updated within existing Submodels (e.g., Handover Documentation, Technical Data, or newly instantiated Circularity Submodels [76]).

In the Recovery phase, data relevant for reuse or recycling, such as verified material composition, updated carbon footprint values reflecting avoided emissions, and quality grading for secondary material use, can be incorporated. This dynamic update process is technically supported through standardized AAS service interfaces (e.g., REST-based APIs) [52], enabling authorized stakeholders (e.g., demolition contractors, recyclers, or certification bodies) to append, validate, or modify SubmodelElements while preserving semantic consistency via semanticIds [77]. Versioning ensures traceability of modifications over time, thereby maintaining data integrity throughout multiple life cycles.

Through this lifecycle-aware modelling approach, the DPP remains an evolving information container that accompanies the concrete element beyond its initial installation, thereby operationalizing circular economy principles in alignment with emerging DPP regulations.

## Investigating consumer perceptions of a DPP in a psychological study

The aim of the psychological study was to gain insight into the consumer perceptions of concrete stair elements presented with DPPs using an exemplary consumer group. The study sought to investigate the general interest of consumers in additional information about a more resource-efficient concrete alternative, and the effect of this additional information presented via a DPP on the consumers’ perceptions of the concrete element.

### Description of the methodology

A two-part online study was conducted via LimeSurvey [78] in German language. In the first part of the study, consumers rated an RAC stair element without being provided any additional information and indicated their interest in additional information about the product. In a second part of the survey, they completed a scenario-based survey [79], which allows the investigation of the causal effect of different component properties (material, environmental impact and structural performance) presented via a DPP on the consumers’ perceptions.

#### DPP mock-up.

**Fig 5:**
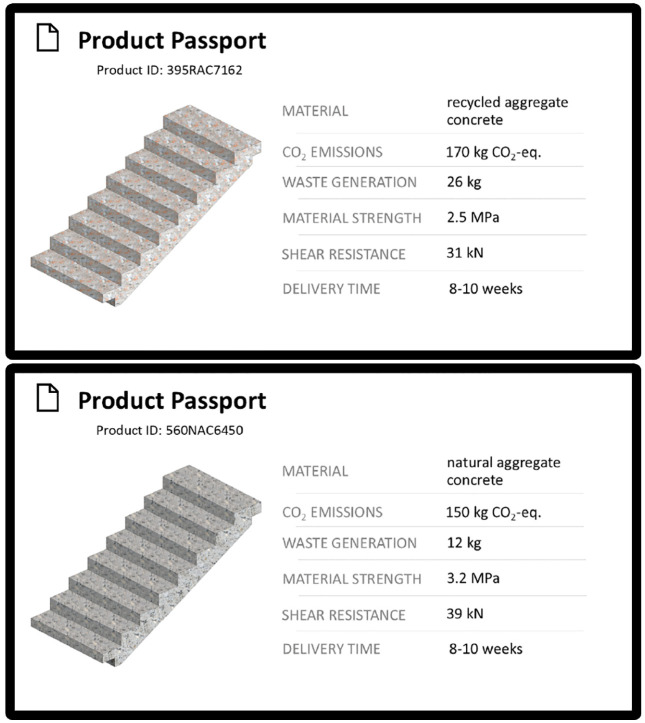
DPP-mockups. Translated version of a DPP-mockup presented to participants, including information on the material, CO_2_-emissions, waste generation, material strength and shear resistance. One DPP for a recycled aggregate concrete element with high environmental impact and reduced structural performance (above), and one DPP for a natural aggregate concrete element with reduced environmental impact and high structural performance (below) represents the real data. Only one DPP was presented at a time.

The previously implemented DPPs were used to develop a series of mock-ups for the assessment of the consumers’ perceptions. These mock-ups were created to simulate the design of DPPs, including a picture of the relevant product, its attributes, and their specific characteristics. In order to avoid overwhelming the participants, a selection of five attributes was made out of the three previously defined categories “Material Composition”, “Environmental Impact”, and “Performance and Safety” (see Section ”Implementation and results”). To assess the impact of the information within DPPs on how the material is perceived, the attribute characteristics for each category were systematically varied. The “Material Composition” was varied between natural aggregate concrete (NAC) and recycled aggregate concrete (RAC). The “Environmental Impact” was varied between low and high based on CO_2_-emissions (150 kg CO_2_-eq. vs. 170 kg CO_2_-eq.) and waste generation (12 kg vs. 26 kg). “Performance and Safety” was varied between low and high based on material strength (concrete tensile strength: 2.5 MPa vs. 3.2 MPa) and shear resistance (31 kN vs. 39 kN), indicating the load level leading to first cracks as well as the calculated failure. The selected values were derived from the technical data sets of a staircase element constructed with either RAC or NAC (see Section “Implementation and results”). The attributes were selected based on the availability of technical data, real differences between NAC and RAC within the attributes and a higher comprehensibility compared to similar attributes. To further increase clarity, a short description of the properties was added. Two cases of a RAC and NAC staircase are presented below as presented to the participants (Fig 5).

#### Procedure and measurements.

Eighty-three participants (90.36% female, *M*_age_ = 21, *SD*_age_ = 2.6, Range_age_ = 18–31) completed the study. Most participants did not have prior knowledge (96.39% of participants), and a few participants reported limited prior knowledge (3.61% of participants) of the construction industry. The mean environmental consciousness was high, with *M* = 4.02 (*SD* = 0.61) out of five. For the second part of the study, participants indicated a mean confidence in their answers of *M* = 3.6 (*SD* = 1.69) out of 7. There was no monetary compensation, but psychology students had the option to gain course credit for their participation.

All participants completed the study with the following procedure. After being informed about the study, accepting the data protection form and giving their written informed consent, participants were asked to image themselves as someone currently building their own home and planning to buy a stair element, made either from RAC or NAC. Participants rated their interest in additional information for the RAC and the NAC stair element, and their willingness to pay [10], perceived environmental value [80] and perceived functional risk [80] for the RAC stair element. Additionally, participants had to choose one of the two types of stair elements. They could then mark all attributes they would be interested in out of a list of all collected product attributes, separated by the five categories “Product Identification and Traceability”, “Material Composition”, “Environmental Impact”, “Performance and Safety”, and “Circularity and End-of-Life” An open answer field was included for additional comments.

At the start of the second part of the study, participants were informed that they would be presented with eight different stair models, each described with the same attributes. A simple explanation of all attributes was given during the rating of the stair models, which were presented via the eight previously described DPP mock-ups in a randomized order. For each of the stair elements, participants rated their willingness to pay, perceived environmental value, perceived functional and product preference. After rating all eight elements, participants indicated their confidence in their answers and were provided with the option to give feedback to the presented DPPs. Then, their environmental consciousness was measured with a nine-item questionnaire based on [81] as a control variable, as previous studies showed that the acceptance of sustainable alternatives depends on the environmental attitudes [19,82–85]. Finally, basic demographic information like age, gender, prior knowledge and field of study was queried. The whole study lasted approximately 15 minutes. Items of all dependent variables can be found in the Appendix, S1 Table A1. Descriptive statistics for the perceptions of the eight vignette scenarios can be found in the Appendix, S2 Table A2.

## Results

### Perceptions of stair elements without DPP

For the perception of a RAC stair element without additional information, participants rated the perceived environmental value as high, with *M* = 5.89 out of seven, and the perceived functional risk as low, with *M* = 2.56 out of seven. They were willing to pay *M* = 1119.04 € (*SD* = 258.53 €), which did significantly differ from the default value of 1000 € provided for NAC elements, *t*(82) = 4.19, *p* < .001. Generally, participants were interested in additional information, but more so for the RAC element (*M* = 6.13) compared to the NAC element (*M* = 4.07), *t*(82) = −9.83, *p* < .001. Addi*t*ionally, the overwhelming majority of participants chose the RAC stair element (81.93%) compared to the NAC stair element (18.07%), *X*^2^(1) = 65.16, *p* < .001, *h* = 1.38, which indicates a large effect size [86]. When choosing which attributes they would be interested in out of a list of all collected attributes, 97.6% chose *Hazardous materials*, followed by *Structural Performance* (95.2%) and *Manufacturing description* (92.8%). Only 25.3% chose *Product identifier*, 21.7% chose *Ozone Depletion Potential* and 16.9% chose *Eutrophication Potential*. For an overview of the attributes, see Appendix, S3 Table A3.

### Perceptions of stair elements with DPP

To investigate the effect of different component properties on participants’ perceptions, results of the scenario-based survey are analyzed next. To this end, four 2 (material: RAC vs. NAC) x 2 (environmental impact: low vs. high) x 2 (structural performance: high vs. low) repeated measures Analyses of Variance (ANOVAs) were calculated for each of the dependent variables perceived environmental value, perceived functional risk, willingness to pay and product preference.

**Fig 6:**
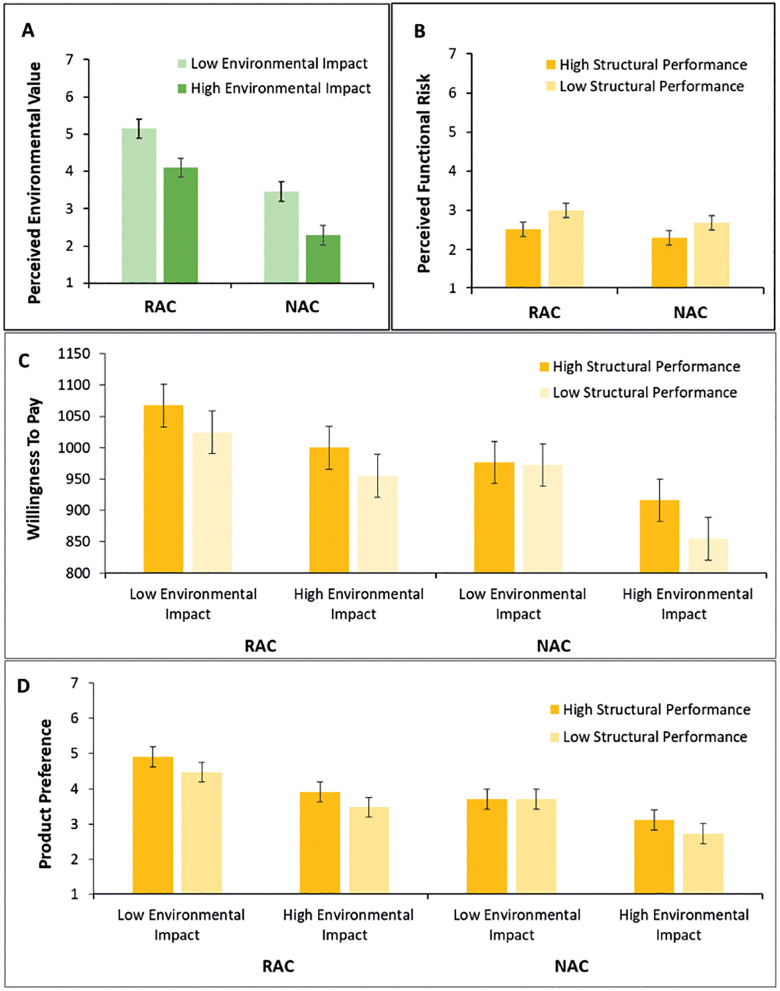
Results of the psychological study on the DPP consumer perception. Figure A shows mean scores for *Perceived Environmental Value* for recycled aggregate concrete (RAC) and natural aggregate concrete (NAC) dependent on the environmental impact averaged over structural performance; Figure B shows mean scores for *Perceived Functional Risk* for RAC and NAC dependent on the structural performance averaged over environmental impact; Figure C shows *Willingness To Pay* for RAC and NAC dependent on the environmental impact and the structural performance; Figure D shows mean scores for *Product Preference* for RAC and NAC dependent on the environmental impact and the structural performance. Brackets indicate confidence intervals, calculated based on the pooled mean square errors following the method described by [87].

First, as shown in Fig 6 A, perceived environmental value was significantly higher for RAC stair elements (vs. NAC) and for stair elements with a low environmental impact (vs. a high environmental impact). The main effect of structural performance was not significant (*p* > .05). Second, perceived functional risk (Fig 6 B) was significantly higher for RAC stair elements (vs. NAC) and for stair elements with a low structural performance (vs. a high structural performance). The main effect of environmental impact was non-significant (*p* > .05). Third, willingness to pay (Fig 6 C) was significantly higher for RAC stair elements (vs. NAC), for stair elements with a low environmental impact (vs. a high environmental impact) and for stair elements with a high structural performance (vs. a low structural performance). Lastly, product preference (Fig 6 D) was significantly higher for RAC stair elements (vs. NAC), for stair elements with a low environmental impact (vs. a high environmental impact) and for stair elements with a high structural performance (vs. a low structural performance). All interactions were non-significant (all *p* > .05). An overview of the effects can be found in Table 1.

**Table 1:** Results of the four ANOVAs for the effects of Material, Environmental Impact and Structural Performance on the four dependent variables Perceived Environmental Value (PEV), Perceived Functional Risk (PFR), Willingness to Pay (WTP) and Product Preference (PP), presented with Means (*M*) and Standard Deviations (*SD*) in brackets below.

	Effect								
	Material			Environmental impact			Structural Performance		
	NAC *M* (*SD*)	RAC *M* (*SD*)	*F*(1, 82)	Low *M* (*SD*)	High *M* (*SD*)	*F*(1, 82)	Low *M* (*SD*)	High *M* (*SD*)	*F*(1, 82)
PEV	2.87 (1.35)	4.62 (1.33)	109.01***	4.3 (1.33)	3.19 (1.35)	119.94***	3.76 (1.32)	3.74 (1.36)	0.05
PFR	2.5 (1.11)	2.76 (1.15)	10.57**	2.65 (1.11)	2.61 (1.15)	0.49	2.84 (1.21)	2.41 (1.04)	23.43***
WTP [€]	930 (203)	1012 (217)	15.16***	1010 (203)	932 (217)	30.95***	952 (214)	990 (206)	15.86***
PP	3.32 (1.42)	4.19 (1.50)	32.37***	4.2 (1.41)	3.31 (1.51)	57.14***	3.6 (1.46)	3.91 (1.46)	10.63**

Notes. *F* indicates the *F*-statistics, measuring the influence strength; asterisks confirm statistical significance with * *p* < .05; ** *p* < .01; *** *p* < .001.

### Comparison of RAC stair elements with and without DPP

**Fig 7:**
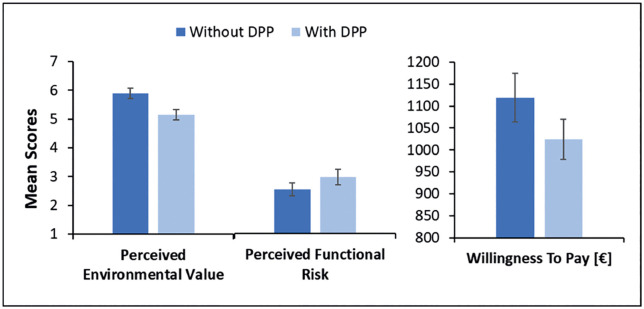
Selected results of the comparison between the case with and without DPP presentation. Mean scores for *Perceived Environmental Value* (left), *Perceived Functional Risk* (middle) and *Willingness To Pay* (right) for the recycled aggregate concrete (RAC) element presented with a Digital Product Pass (DPP) or without. Brackets indicate confidence intervals, calculated based on the pooled mean square errors following the method described by [87].

Lastly, the RAC element presented in the first part of the study without a DPP was compared to an RAC element presented with a DPP via dependent samples *t*-tests. To this end, the RAC element with realistic values (a low environmental impact and a lower structural performance) was chosen. For the RAC element presented without a DPP compared to the RAC element presented with a DPP perceived environmental value was significantly higher, *t*(82) = 5.02, *p* < .001, perceived functional risk was significantly lower, *t*(82) = −3.03, *p* = 0.003 and willingness *t*o pay was significantly higher, *t*(82) = 3.71, *p* < .001. The mean scores for “Perceived Environmen*t*al Value” and “Perceived Functional Risk” as well as “Willingness to Pay” for the RAC element without and with a DPP are presented in Fig 7.

## Discussion

The psychological study was conducted to gain first exploratory insights into consumer perceptions of concrete stair elements presented via DPPs using an exemplary consumer group. The study revealed three main contributions:

First, the participants’ perceptions of a RAC stair element presented without a DPP were generally favorable, as indicated by a significantly higher willingness to pay than the default value set for an NAC stair element. The interest in additional information was also significantly higher for the RAC compared to the NAC element. These results are somehow unexpected, as most studies show a reduced willingness to pay for recycled compared to conventional products [10,20,22]. However, the participants were generally young and had a high environmental consciousness, which has been shown to impact the evaluation and purchase intention of recycled products [19,88]. Additionally, the experiment only posed a hypothetical scenario and not a real purchase, which might result in overly positive attitudes [23,24].

Second, different DPPs were rated, and results show a significant effect of the material, the environmental impact and the structural performance on the participants’ perceptions. Expectedly, a lower environmental impact increased the perceived environmental value, and a higher structural performance decreased perceived functional risk. Interestingly, the material of the aggregates affected how environmental value and risk were perceived, regardless of the objective environmental and structural performance characteristics. Supported by the DPP, the RAC component was perceived to have increased environmental value compared to the NAC component with the same environmental impact. Similarly, the RAC component was perceived to have increased functional risk compared to the NAC component with the same structural performance. These results could imply that consumers expect recycled materials to have environmental benefits that exceed the presented reduction in CO_2_ emissions and waste generation, as well as functional drawbacks that exceed the presented reduction in material strength and shear resistance. On the one hand, a positive bias towards the environmental benefits of RAC seems to be present, which is in line with the recycling bias observed before, describing an overestimation of the effects of recycling [18]. On the other hand, a negative bias towards the functional properties of RAC seems to be present, consistent with previous research [9,23,89]. These biases in consumer perception can lead to unintended consumer behavior [90], and are possible explanations for the present results. The label of material can thus both inform and distort the perception of consumers. The effects of the three properties occurred without statistically significant interaction effects, as indicated by non-significant interactions (all *p* > .05), providing no evidence that the effects of material, environmental impact, and structural performance depended on each other. This means that there was no statistical indication that the effect of one characteristic varied as a function of another characteristic, e.g., the effect of structural performance did not differ between low and high environmental impact conditions.

Third, comparing the consumers’ perceptions of the RAC element presented without a DPP and the one presented with a DPP supports this assumption. Contradictory to previous studies [7,10,11], the stair element presented without a DPP was perceived as more favorable regarding perceived environmental value, functional risk and willingness to pay compared to the element presented with a DPP. These findings could be the result of the aforementioned biases. Since both components that were compared in this analysis were recycled elements, the recycling bias [18] could have led participants to overestimate the benefits of recycled aggregate concrete. This estimate may have been greater than what was communicated in the DPP, resulting in a downward correction for the perceived environmental value after receiving the objective data. It is also possible that the finding has a methodological explanation, since adding information via a DPP could have led to information overload, thereby decreasing decision accuracy [91]. However, since the results consistently show a less favorable judgement for the RAC component presented with additional information compared to the one without additional information, it is more likely that participants were more cautious in their judgement after receiving more information, resulting in reduced perceived environmental value, increased functional risk and, as a result, reduced willingness to pay. Future studies could address this finding by using a between-subjects design, where both components can be judged by different participants instead of the same, making relative estimations impossible.

The current study presents valuable insights into the perception of objective properties of stair elements presented via DPPs. The key limitation is the use of a layperson sample. However, we chose this sample for the following reasons: The study was conceptualized as an initial exploration, for which practicality was important. While the use of laypersons may limit generalizability, it was particularly useful for the scope of the present study. Still, it may be valuable for future studies to include a sample of field experts (e.g., architects, construction engineers) to expand the results. However, the effects of interest were cognitive biases, which result out of fundamental limits of information processing and can occur independently of expertise [15–17]. It is therefore likely that biases similar to those observed in this study also occur in other populations and for a range of use cases. Future studies could additionally expand the results to different building components. While similar effects of the examined material properties are to be expected, it is possible that the importance of risk perception for the general acceptance might be higher for structurally critical components, e.g., the roof, compared to, e.g., a non-loadbearing wall.

To summarize, the findings suggest that consumer decision-making is influenced not only by objective material characteristics but also by biases in information processing, including exaggerated perceptions of environmental benefits and functional risks for recycled materials. These insights highlight the importance of addressing cognitive biases and optimizing information presentation in DPPs to enhance informed decision-making and acceptance of sustainable innovations.

## Conclusion and outlook

The current project highlights the potential of Digital Product Passports (DPPs) to promote a circular economy within the construction industry, particularly by promoting the adoption of recycled aggregate concrete (RAC). The recycling of construction waste is dependent on transparent information about the used material, for which the DPP provides a solution by enabling the collection and exchange of product-related data. We created a DPP based on international standards of the Asset Administration Shell (AAS) using the example of an NAC and RAC stair element. The psychological study revealed that potential consumers were sensitive to variations in DPP content, and while they viewed RAC as more environmentally friendly, they also attributed a higher risk to it, regardless of the objective properties of the material communicated via the DPP. Interestingly, the presentation of some information via a DPP negatively impacted how the product was perceived, implicating that receiving detailed objective properties can result to more cautious judgements. This result reveals a need for careful presentation of information [92]. Further research could examine a dynamic presentation of pre-selected attributes depending on the target group, as the presentation of certain information could influence their perception of the product in different ways, thus supporting the optimized use of DPPs. While manufacturers and recycling companies will need detailed information with technical data, consumers’ informed decision-making might be fostered by presenting technical data supported by visual guidelines, e.g., by using gauges to present compiled data and recommendations. These recommendations could also help reduce consumers’ biases in decision making by highlighting the actual benefits and disadvantages of RAC. For example, the objectively reduced structural performance of RAC compared to NAC is negligible in their actual use, since both concrete elements are subject to strict guidelines and rather conservative rules regarding material strength and similar properties. A visual indicator of the RAC’s structural performance still upholding standards and being rated as “as good” as the NAC’s structural performance might help consumers more accurately assess the materials properties.

Ultimately, the current study illustrates that the development of a DPP and its implementation for construction elements is feasible using the AAS, and that the way in which information is presented using a DPP can impact consumer perceptions. The developed DPP could be applied to more complex building components such as different variants of concrete floor slabs (e.g., prestressed hollow-core slabs vs. semi-prefabricated lattice girder slabs) in future studies. For a holistic approach, these studies should include the consideration of psychological perceptions, especially with regard to increased technical constraints and interdependencies. By advancing research in this area, we can make an important contribution to building a circular economy in the construction industry, which is an essential step towards reducing greenhouse gas emissions and resource depletion.

## Supporting information

S1 Table A1 Items for the measurements in the study presented with Means (*M*) and Standard Deviations (*SD*) in brackets. (DOCX)

S2 Table A2 Descriptive statistics presented with Means (*M*) and Standard Deviations (*SD*) for the four dependent variables Perceived Environmental Value, Perceived Functional Risk, Willingness to Pay and Product Preference, calculated for each of the eight DPPs with either Recycled Aggregate Concrete (RAC) or Natural Aggregate Concrete (NAC), low or high Environmental Impact (ENV) and low or high Structural Performance (STR). (DOCX)

S3 Table A3 Attributes chosen by participants to indicate their interest in more information; Multiple choice was possible. (DOCX)

S1 File RePass data and R script for evaluation of the psychological study. (ZIP)
