# Supplementary material for: Design and acceptance assessment of a digital product passport for recycled and natural aggregate concrete elements
Source: PLoS One. 2026 Apr 20;21(4):e0347562. doi: 10.1371/journal.pone.0347562 (PMC13095097; doi:10.1371/journal.pone.0347562)
Supplement: S1 Table A1 — (DOCX) [file pone.0347562.s001.docx]

S1 Table A1 Items for the measurements in the study presented with Means (*M*) and Standard Deviations (*SD*) in brackets.

| Interest in additional information^1^ | | *M* (*SD*) |  |
| --- | --- | --- | --- |
|  | - I would like to have more information about the stairs made of conventional concrete  - I would like to have more information about the stairs made of recycled concrete | 4.07 (1.88)  6.13 (1.11) |  |
| Willingness to pay^2^ | |  |  |
|  | - How much would you be willing to pay for [this product]? | 970.81 (150.47) |  |
| Perceived environmental value^1^ | |  |  |
|  | - [This product] helps save resources. | 3.80 (0.83) |  |
|  | - [This product] has a positive impact on the environment in that it extends the life of discarded materials. | 3.75 (0.75) |  |
|  | - [This product] is environmentally friendly. | 3.65 (0.89) |  |
|  | - [This product] has more environmental benefits than other [products]. | 3.79 (0.82) |  |
| Perceived functional risk^1^ | |  |  |
|  | - [This product] would not be durable. | 2.58 (0.92) |  |
|  | - I won’t be able to use [this product] for a long time. | 2.49 (0.90) |  |
|  | - [This product] is likely to wear out faster than general [product]. | 2.56 (0.85) |  |
|  | - [This product] is likely to sustain damage over time. | 2.88 (1.04) |  |
| Product preference^1^ | |  |  |
|  | - I prefer this staircase | 3.15 (0.55) |  |
| Confidence^1^ | |  |  |
|  | - I am confident in my assessments | 3.60 (1.69) |  |
| *Notes*. [Product] was replaced by the relevant type of staircase  ^1^ answered on a 7-point scale from 1 (strongly disagree) to 7 (strongly agree)  ^2^ answered on a sliding bar from 0 € to 2000 €, with a default set at 1000 € | |  |  |
