# Supplementary material for: Design and acceptance assessment of a digital product passport for recycled and natural aggregate concrete elements
Source: PLoS One. 2026 Apr 20;21(4):e0347562. doi: 10.1371/journal.pone.0347562 (PMC13095097; doi:10.1371/journal.pone.0347562)
Supplement: S2 Table A2 — (DOCX) [file pone.0347562.s002.docx]

S2 Table A2 Descriptive statistics presented with Means (*M*) and Standard Deviations (*SD*) for the four dependent variables Perceived Environmental Value, Perceived Functional Risk, Willingness to Pay and Product Preference, calculated for each of the eight DPPs with either Recycled Aggregate Concrete (RAC) or Natural Aggregate Concrete (NAC), low or high Environmental Impact (ENV) and low or high Structural Performance (STR).

|  | Perceived Environmental Value^1^ | | Perceived Functional Risk^1^ | | Willingness to pay² | | | Product Preference^1^ | | |
| --- | --- | --- | --- | --- | --- | --- | --- | --- | --- | --- |
|  | *M* | *SD* | *M* | *SD* | *M* | | *SD* | *M* | | *SD* |
| RAC lowENV lowSTR | 5.16 | 1.25 | 3.00 | 1.24 | 1024.46 | 213.76 | | 4.47 | 1.48 | |
| RAC lowENV highSTR | 5.13 | 1.16 | 2.52 | 1.04 | 1067.35 | 217.47 | | 4.90 | 1.30 | |
| RAC highENV lowSTR | 4.23 | 1.42 | 3.02 | 1.28 | 955.13 | 214.40 | | 3.48 | 1.58 | |
| RAC highENV highSTR | 3.97 | 1.52 | 2.52 | 1.05 | 999.92 | 224.29 | | 3.92 | 1.62 | |
| NAC lowENV lowSTR | 3.44 | 1.43 | 2.74 | 1.10 | 972.13 | 199.13 | | 3.71 | 1.39 | |
| NAC lowENV highSTR | 3.48 | 1.50 | 2.33 | 1.05 | 976.61 | 183.16 | | 3.71 | 1.46 | |
| NAC highENV lowSTR | 2.20 | 1.22 | 2.62 | 1.23 | 854.67 | 229.53 | | 2.73 | 1.39 | |
| NAC highENV highSTR | 2.38 | 1.25 | 2.29 | 1.04 | 916.23 | 199.53 | | 3.12 | 1.45 | |
| *Note*:  ^1^ answered on a 7-point scale from 1 (strongly disagree) to 7 (strongly agree)  ^2^ answered on a sliding bar from 0 € to 2000 €, with a default set at 1000 € | | | | | | | | | | |
