# Supplementary material for: Design and acceptance assessment of a digital product passport for recycled and natural aggregate concrete elements
Source: PLoS One. 2026 Apr 20;21(4):e0347562. doi: 10.1371/journal.pone.0347562 (PMC13095097; doi:10.1371/journal.pone.0347562)
Supplement: S3 Table A3 — (DOCX) [file pone.0347562.s003.docx]

S3 Table A3 Attributes chosen by participants to indicate their interest in more information; Multiple choice was possible.

| Attributes | Category | Frequency |
| --- | --- | --- |
| Hazardous materials | Material Composition | 81 |
| Structural Performance | Performance and Safety | 79 |
| Manufacturing description | Product Identification and Traceability | 77 |
| Material properties | Performance and Safety | 76 |
| Product description | Product Identification and Traceability | 75 |
| Durability Metrics | Performance and Safety | 73 |
| Concrete Composition | Material Composition | 72 |
| Energy Consumption | Environmental Impact | 72 |
| Resource depletion | Environmental Impact | 72 |
| Supply chain traceability | Product Identification and Traceability | 64 |
| Global Warming Potential | Environmental Impact | 62 |
| Fatigue Resistance | Performance and Safety | 62 |
| Reuse Potential | Circularity and End-of-Life | 61 |
| Fire Resistance | Performance and Safety | 60 |
| Air Pollution | Environmental Impact | 58 |
| Disassembly instructions | Circularity and End-of-Life | 57 |
| Reinforcement | Material Composition | 50 |
| Waste management recommendations | Circularity and End-of-Life | 49 |
| Land Use | Environmental Impact | 26 |
| Type of concrete aggregates | Material Composition | 23 |
| Acidification Potential | Environmental Impact | 23 |
| Product identifier | Product Identification and Traceability | 21 |
| Ozone Depletion Potential | Environmental Impact | 18 |
| Eutrophication Potential | Environmental Impact | 14 |
